# Osteogenic induction of menstrual blood mesenchymal stem cell by different *Ferula* species extracts

**Published:** 2021

**Authors:** Razieh Dalirfardouei, Elahe Mahdipour, Mehrdad Iranshahi, Khadijeh Jamialahmadi

**Affiliations:** 1 *Research Center for Molecular Medicine, Hamadan University of Medical Sciences, Hamadan, Iran*; 2 *Department of Medical Biotechnology and Nanotechnology, Faculty of Medicine, Mashhad University of Medical Sciences, Mashhad, Iran*; 3 *Biotechnology Research Center, Pharmaceutical Technology Institute, Mashhad University of Medical Sciences, Mashhad, Iran*

**Keywords:** Medicinal plant, Ferula species, Osteogenic differentiation, ALP activity, Runx-2, Mesenchymal stem cells

## Abstract

**Objective:**

*Ferula* spp. have many applications in complementary medicine and are recognized as the most important sources of natural products for bone health and regeneration especially in postmenopausal women. Therefore, the aim of this study was to investigate the effects of the extracts from three *Ferula *species on proliferation and osteogenesis potential of human menstrual blood-derived mesenchymal stem cells (MenSCs).

**Materials and Methods:**

The possible cytotoxic activity of three members of *Ferula *spp. (at concentrations of 5 to 100 μg/ml) was determined using MTT (3-(4, 5-dimethylthiazol-2-yl)-2, 5-diphenyl tetrazolium bromide) assay. Alkaline phosphatase (ALP) activity assay, Alizarin Red-S staining, and the expression analysis of an osteoblastic gene were performed to evaluate osteogenic differentiation potential.

**Results:**

The extracts of *F. flabelliloba *and* F. szowitsiana *decreased the viability and growth of MenSCs while *F. foetida *increased the proliferation of cells after 72 hr incubation. Treatment of MenSCs with selected plant extracts revealed that *F. foetida* and *F. szowitsiana *could enhance the osteogenic potential of MenSCs in terms of ALP activity. The *Runx-2* expression in the presence of *F. foetida* was significantly greater than observed following treatment with 17β-estradiol (as positive control).

**Conclusion:**

The results of this study suggest that *F. foetida* and *F. szowitsiana* may have therapeutic values as a nutraceutical with respect to their considerable influence on osteogenic potential of mesenchymal stem cells.

## Introduction

Osteoporosis is a major public health issue among the elderly population especially postmenopausal women; under this condition, the skeleton becomes fragile, and risk of fractures increases. According to Iran’s ministry of health statistics, 33% of women and 20% of men over 50 years old suffer from osteoporosis (Mirhashemi et al., 2017 ▶). By 2050, the global incidence of osteoporotic fractures has been predicted to reach 6.26 million (Cole et al., 2008 ▶). The most common therapeutic regimen for recovery of postmenopausal bone loss is estrogen replacement therapy; however, it is accompanied by well-known negative side effects including increased risk of cardiovascular diseases and malignant breast, ovarian, and endometrial tumors (Beral, 2003 ▶; Carla Palumbo et al., 2012 ▶; Collaborative Group on Epidemiological Studies of Ovarian, 2015 ▶; Salaminia et al., 2019 ▶). 

In recent years, there has been considerable interest in using medicinal plants to develop an effective osteoporosis treatment with minimal clinical complications. *Ferula *L*.* belongs to the Apiaceae family (Division: Magnoliophytaand Order: Apiales) and is the most commonly investigated medicinal herb for treatment of different diseases (Iranshahy and Iranshahi, 2011 ▶; Safaeian et al., 2015 ▶). *Ferula *L. including *F. assa-foetida*, *F. gummosa*, and *F. persica*, are distributed in the center, northern, and southern parts of Iran. They comprise the largest genus of Apiaceae in Iran (Moazzami et al., 2018 ▶). Phytochemical analysis has identified that *Ferula *spp*.* are rich sources of biologically active ingredients such as phytoestrogen compounds like isoflavon and sesquiterpene ester (Akaberi et al., 2015 ▶; Saeedet al., 2012 ▶). Many *in vivo* studies have widely investigated the anti-osteoporotic effects of phytoestrogens, plant-based estrogen, such as ferutinin (Palumbo et al., 2012 ▶; Cavani et al., 2012 ▶; Ferretti et al., 2010 ▶; Palumbo et al., 2009 ▶), and revealed similar preventive or recovery effects on bone mass compared to estradiol benzoate in ovariectomized rats. Unlike estrogen, ferutinin, the most widely studied phytoestrogen found in some *Ferula* genus, had cytotoxic and apoptotic effects on breast cancer cell lines (Matinet al., 2014 ▶; Safiet al., 2018 ▶) suggesting this natural compound as a promising alternative to hormone replacement therapy. 

Although *Ferula* spp. have a long history in traditional medicine in Iran, little is known on the effects of the crude extract of* Ferula* species on bone repair. Therefore, the aim of this study was to explore the osteogenic stimulatory potential of the methanolic-dichloromethane extracts of three different *Ferula* species including *Ferula flabelliloba, Ferula foetida*, and *Ferula szowitsiana*. In the present study, we investigated the ability of the plant extracts to modulate the osteogenesis capacity of human menstrual blood-derived mesenchymal stem cells (MenSCs). Osteoblastic differentiation was evaluated with regard to alkaline phosphatase (ALP) activity and gene expression of an osteogenesis-related marker. Mineralization and calcium production were also assessed by Alizarin Red-S staining. 

## Materials and Methods

**Chemicals, reagents, and antibodies**

For cell culture and differentiation induction, fetal bovine serum (FBS) and Minimum Essential Medium Eagle alpha (α-MEM) were purchased from Gibco (Gibco, USA) and Biowest (Biowest, France), respectively. Adipogenic and osteogenic differentiation media, and PBS (phosphate-buffered saline) were acquired from Invitrogen (Invitrogen; Thermo Fisher Scientific, Inc.). Alizarin Red-S, Oil Red O, dimethylsulfoxide (DMSO), 3-(4, 5-dimethyl-thiazol-2yl)-2, 5-diphenyl tetrazolium bromide (MTT), and estradiol, were obtained from Merck (Merck, Germany). The primary antibodies including CD29, CD90, CD117, and HLA-DR were purchased from Santa Cruz Biotech (Santa Cruz Biotech, USA). Hoechst 33258 and protease inhibitor cocktail were obtained from Sigma-Aldrich. Trypsin from BioIDEA (Iran), alkaline phosphatase (ALP) colorimetric assay kit from Parsazmoon (Iran), and bisinconinic acid (BCA) kit from Parstous (Iran) were acquired. RiboEx solution and RevertAid First Strand cDNA synthesis kit were purchased from GENEALL (GENEALL, Korea) and Fermentase (K1622, Fermentase Life Sciences), respectively.

**Preparing of plant extract**

The roots of *F. flabelliloba* were collected from the Hezarmasjed mountains, Khorasan Razavi province, Iran, in April 2006. Also, *F. foetida*was collected from the mountains of Dorouneh village, Khorasan Razavi province, Iran, in May 2012. Both of them were identified by Mohammad Reza Joharchi, Ferdowsi University of Mashhad Herbarium (FUMH). The roots of *F. szowitsiana* were collected from the mountains of Golestan forest, Golestan province, Iran, in June 2003 and identified by Dr. Hossein Akhani, Department of Botany, Faculty of Sciences, Tehran University. Voucher specimens of the plants (No. 1004, No. 12612 and No. M1001, respectively) were deposited in the herbarium of the School of Pharmacy, Mashhad University of Medical Sciences, Mashhad, Iran. Fresh roots of the plants (250 g) were powdered and extracted by methanol using the maceration method at room temperature. Then, the extracts were concentrated under vacuum to yield brownish viscous extracts.

**MSCs isolation and characterization**

MenSCs were obtained from menstrual blood of healthy woman adults (18-30 years of age) as previously described (Dalirfardouei et al., 2018 ▶). All volunteer blood donors signed informed consent form, according to the guideline of the Ethics Committee of Mashhad University of Medical Sciences (Ref No. IR. MUMS REC.1393.961). In brief, human MenSCs were isolated by the method of menstrual whole blood culture in α-MEM supplemented with 15% v/v of FBS, 100 U/ml penicillin and 100 mg/ml streptomycin, and 2.5 μg/ml amphotericin B, and incubated at 37°C in a humidified atmosphere containing 5% v/v CO_2_. The growth medium was changed after 24 hr and then, every 3 days. MenSCsin passage 3 were harvested by trypsinization and characterized using flow cytometry as reported by Dalirfardouei et al. (2019) ▶. Osteogenic and adipogenic differentiation potential in MenSCs was examined by culturing the cells in favorable conditions for adipogenesis or osteogenesis as described in our previous studies (Dalirfardouei et al., 2018 ▶; Dalirfardouei et al., 2019 ▶). 

**MTT cell viability assay**

To evaluate the cytotoxicity of different plant extracts, MenSCs at passage 4 were suspended in the medium (α-MEM with 15% v/v FBS) and then, seeded into a 96-well plate at a density of 1000 cells/well. After 24 hr, the plant extracts (solubilized in DMSO) at concentrations ranging from 5 to 100 μg/ml or 0.1% DMSO (as a vehicle control) were added to the growth medium (n=5 repeats for each concentration). After 24, 48 and 72 hr of incubation, cell viability was determined using the MTT assay. To do so, 10 μl of 5 mg/ml MTT solution was added to each well and incubated at 37°C. After 5 hr, the medium was removed and the formazan crystals were dissolved in 100 μl of DMSO. The absorbance of each well was measured at 570 nm. Three independent MTT assay was performed for each plant extract (Riss et al., 2013 ▶).

**MenSCs treatment with plant extracts and experimental groups**

MenSCs at passage 4 were plated at a density of 10^4^ cells/well in a 24-well plate and allowed to grow. At 70-80% confluency, cells were washed with PBS then incubated with osteogenic differentiation media (Invitrogen) supplemented with different plant extracts at the final concentration of 20 µg/ml or 17β-Estradiol (10^−8^ M) as a positive control. All experimental groups are defined in Table 1. The culture medium was changed every 3-4 days until 14 or 21 days of culture. Osteogenic activity of each plant extracts was then assessed by Alizarin Red-S staining, ALP activity assay, and Runt-related transcription factor 2 (*RUNX2*) gene expression (a key transcription factor associated with osteoblast differentiation).

**Mineralization- Alizarin Red-S staining**

After 21-day treatment of the cells with the plant extracts, MenSCs were stained with Alizarin Red-S to evaluate calcium production (Dalirfardouei et al., 2018 ▶). For fixation, the monolayer cells were incubated with 4% v/v formaldehyde for 20 min at room temperature (R.T). The cells were then incubated with 2% w/v Alizarin Red-S solution (pH 4.1-4.3) for 20 min at R.T. After this period, the cells were washed with distilled water to remove excess dye. Staining was evaluated using a light microscope (Olympus, USA). MenSCs cultured in growth complete α-MEM medium supplemented with DMSO (the vehicle of plant extract) or 17β-Estradiol (10^−8^ M), served as a vehicle and positive control, respectively. The cells producing calcium were uniformly stained red. 

**Alkaline phosphatase activity assay**

After treatment of the MenSCs with different plant extracts for 21 days, cells were washed with PBS and harvested with trypsin-EDTA. After centrifugation at 300 ×g for 10 min at R.T, the cells were lysed in 100 µl of NP40 lysis buffer containing freshly prepared protease inhibitor cocktail. The ALP activity of cell lysates was determined using the alkaline phosphatase colorimetric assay kit according to the manufacturer’s instruction. Briefly, 5 μl of the cell lysate was added to a 96-well plate containing 100 μl assay buffer and 100 μl p-nitrophenyl phosphate (pNPP). The mixtures were incubated at R.T for 1 min, then, the absorbance was measured immediately, and after 1, 2 and 3 min at 405 nm using Epoch^TM^ microplate spectrophotometer (BioTek). 

To calculate the specific activity of the ALP enzyme, the total protein concentration was measured in the cell lysate by the bisinchoninic acid (BCA) protein assay kit (Parstous, Iran) according to the manufacturer’s instruction. The ALP activity of cell lysates was calculated according to the generated ALP standard curve. The ALP specific activity was normalized against the total DNA amount. 

**DNA measurements**

The DNA content was measured using the fluorescent dye Hoechst 33258 in the cell lysate prepared for ALP assay (Brunk et al., 1979 ▶). Briefly, 5 μl of the cell lysate was added to a 96-well black plate containing 265 μl assay buffer (pH 7, 100 mM NaCl, 10 mM EDTA, 10 mM Tris) and 30 μl Hoechst Dye (1 μg/ml). The final concentration of Hoechst Dye was 0.1 μg/ml. The plate was incubated away from light for 5 min. The fluorescent signal was read using a microplate reader (Perkin-Elmer spectrometer) at excitation of 350 nm and emission of 450 nm. The DNA concentration was determined using a known amount of purified human plasma DNA as the standard.

**Table 1:** Differentexperimental groups and their nomenclature

**Components** **Groups**	**Commercial osteogenesis media**	**Plant extract**	**DMSO**	**Estradiol**
**Treatment group**	Present	20 µg/ml	0.1%	-
**Positive group**	Present	-	-	10^-7^ M
**Vehicle group**	-	-	0.1%	-

DMSO, Dimethyl sulfoxide

***Runx-2*** **gene expression analysis**

The total RNA was extracted from the treated MenSCs 14 and 21 days after induction of differentiation using RiboEx solution according to the manufacturer’s instruction. The purity of the total RNA samples was determined by the ratio of absorbance at 260 and 280 nm, and found to be between 1.8 and 2.0. Total cellular RNA samples were treated with DNase I, RNase‐free (EN0521, Thermo Fisher Scientific) to remove genomic DNA fragments. 500 ng of each total RNA was reversed transcribed to cDNA using RevertAid First Strand cDNA synthesis kit with random hexamer according to the manufacturer’s protocol using ABI thermal cycler (Applied Biosystem). 

Gene expression was assessed by quantitative real-time PCR (qRT-PCR) using SYBR® Premix Ex Taq™ 11 (Takara, Japan) in a Roche Lightcycler® 96 instrument with the specific primers for *Runx-2* gene and *B2m* gene used as an endogenous internal control. The analysis of standard curve and the melting curve was performed to assess the quality of quantitative PCR reaction. All primers were designed using Primer-BLAST (www.ncbi.nlm.nih.gov/tools/primer‐blast/) and further analyzed by Gene Runner Version 4.0.9.68 Beta software. The primers used in the present study, are listed in Table 2. The expression level was determined using the REST 2009 software (Relative Expression Software Tool) (Pfaffl et al., 2002 ▶). 

**Statistical analysis**

All data were analyzed using IBM SPSS Statistics 20 software. One-way ANOVA test was used to analyze MTT and ALP activity data followed by Tukey *post-hoc* test to compare different groups. All data are expressed as the mean±SD. The gene expression data were analyzed using the REST software. All charts were drawn by GraphPad Prism 6. The values of p<0.001-0.05 were considered statistically significant.

## Results

**MenSCs characterization**

**Figure 1:**
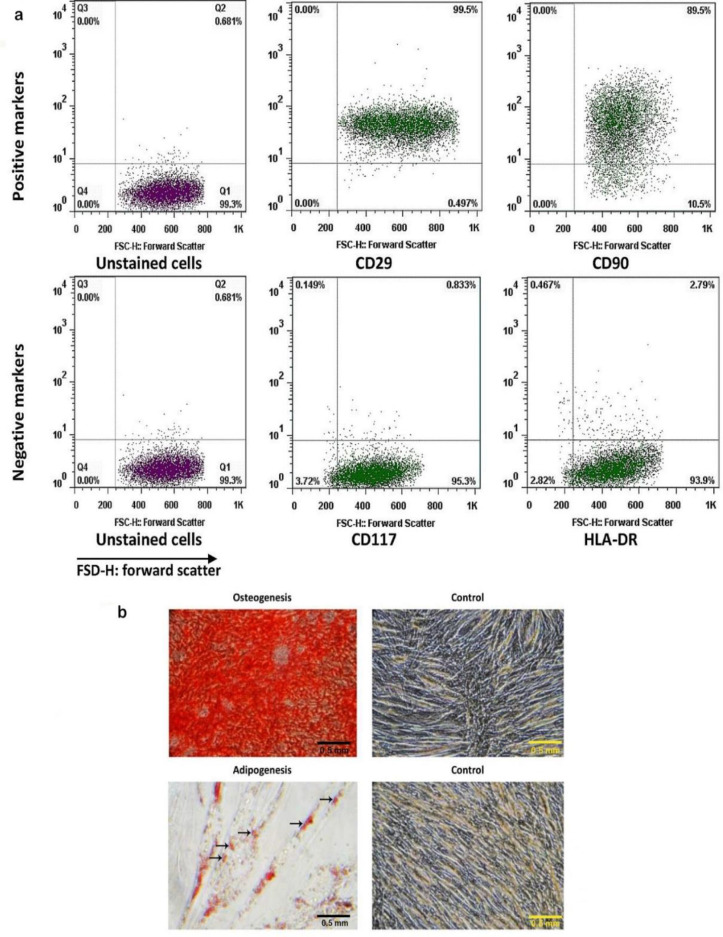
Characterization of MenSCs. (a) Immunophenotyping of cell surface antigens, MenSCs isolated at passage 3 were strongly positive for CD29 and CD90. These cells did not express CD117 and HLA-DR. (b) Differentiation potential, MenSCs at passage 3 could produce calcium and lipid droplet under osteogenesis (top, 400× magnification) and adipogenesis (bottom, 400× magnification) conditions, respectively. The black arrows show the droplet oil inside the cell

The mesenchymal stem cells were isolated from human menstrual blood based on their feature of plastic adherence. The flow cytometer analysis revealed that the spindle-shaped MenSCs expressed stromal markers such as CD29 and CD90 while these cells were negative for hematopoietic progenitor marker CD117 and human major histocompatibility complex class II (HLA-DR) (Figure 1a). Both osteogenic and adipogenic differentiation potential of the MenSCs were evaluated by Alizarin Red-S and Oil Red O staining. The results showed that MenSCs could produce extracellular calcium deposits and lipid vacuoles inside the cells under osteogenic and adipogenic conditions, respectively (Figure 1b).

**MenSCs viability assay**

**Figure 2:**
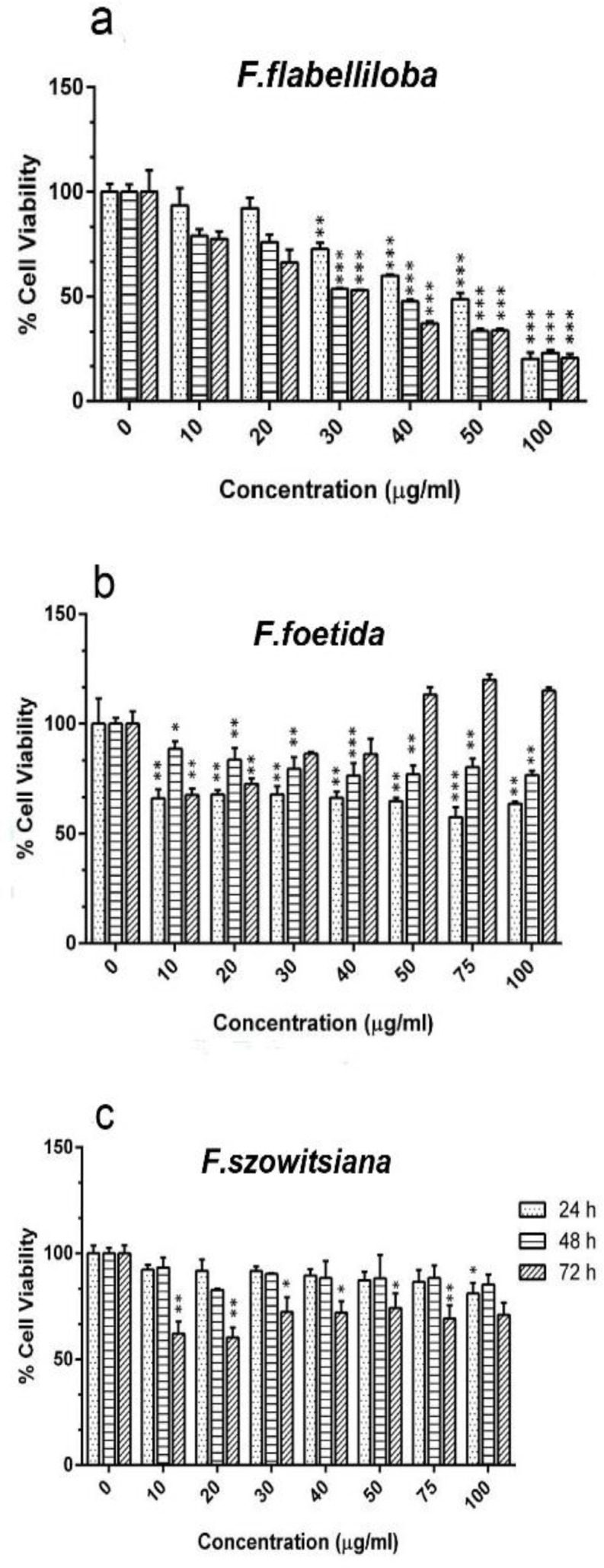
Viability of MenSCs measured by MTT assay. MenSCs at passage 4 were incubated with the extract of (a) *F. flabelliloba*, (b) *F. foetida*, and (c) *F. szowitsiana* at concentrations ranging from 5 to 100 µg/ml. The cell viability was measured 24, 48 and 72 hr after the treatment. All data are expressed as mean±SD. *p<0.05, **p<0.01, and ***P<0.001 show significant differences of each time point in comparison with corresponding time point in the control group).

To evaluate the effect of the three plant extracts on the MenSCs viability by MTT assay, the cells were treated with different concentrations in a range of 5-100 µg/ml for 24, 48 and 72 hr. As shown in Figure 2, only the extraction of *F. flabelliloba* exhibited a concentration- and time-dependent cytotoxic effect on MenSCs (Figure 2a). Although the cell viability of MenSCs decreased 24 and 48 hr post-treatment with* F. foetida*, the enhanced proliferation of these cells was observed 72 hr after treatment with higher concentrations (50-100 µg/ml) (Figure 2b). 

**Table 2:** Gene specific primers used for real time PCR

**Accession number**	**Gene name**	**Forward sequence (5´ to 3´)**	**Reverse sequence (5´ to 3´)**
**NM_001015051.3**	*Runx-2*	GTGGACGAGGCAAGAGTTTC	GCTTCTGTCTGTGCCTTCTG
**NM_009735.3**	*B2m*	ATGCTATCCAGAAACCCCTCA	GGCGGGTGGAACTGTGTTA

The extract of *F. szowitsiana* showed almost equal cytotoxicity toward the MenSCs after 24 and 48 hr. However, its cytotoxicity was remarkably enhanced at 72 hr. According to the MTT results, the highest concentration with the lowest cell toxicity was 20 µg/ml for each plant extract and was used to treat the MenSCs. At this concentration, cell viability was greater than 50% in all treatment groups.

**Calcium deposition and ALP activity**

**Figure 3:**
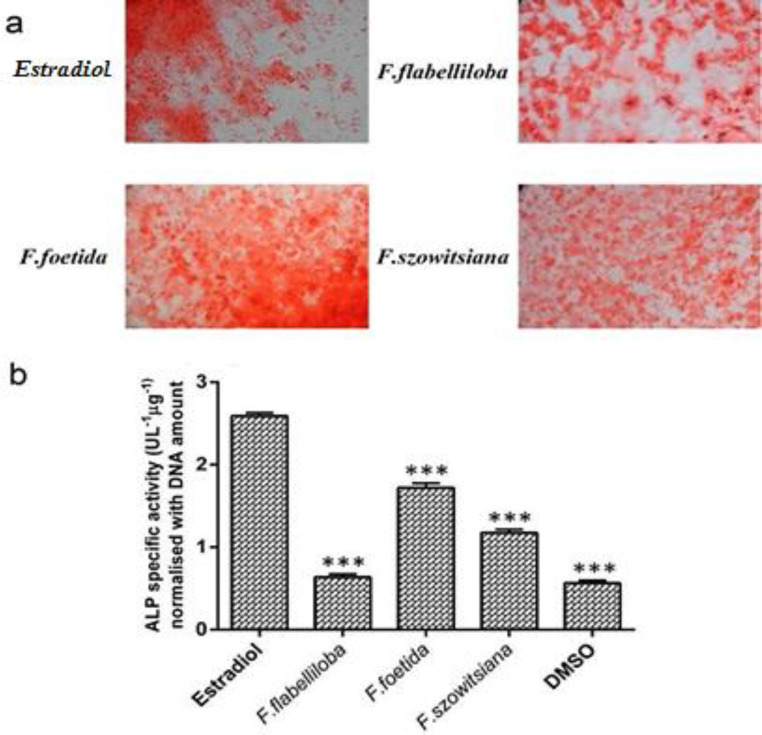
Calcium deposition and alkaline phosphatase activity. (a) The representative images of Alizarin Red-S staining showed the effect of different plant extracts on mineralization and calcium deposition. (b) The specific activity of ALP in the MenSCs treated with the 20 µg/ml of different plant extracts including *F. flabelliloba*, *F. foetida*, *F. szowitsiana*, and estradiol (10^-7^ M, positive control), was determined 21 days after the treatment. All data are expressed as mean±SD. ***p<0.001 show significant differences in comparison with the estradiol-treated group)

To characterize the osteogenic activity of different plant extracts, the production of calcium deposit and the activity of ALP were assessed on day 21 post-treatment. Alizarin Red-S staining showed that the cell culture exposed to 20 µg/ml of each plant extract, could be induced to produce calcium deposits (Figure 3a). The cytosolic ALP activity by MenSCs treated with *F. foetida* was greater than that of the MenSCs treated with *F. flabelliloba* and *F. szowitsiana*. However, the ALP activity was significantly lower in the MenSCs treated with *F. foetida* as compared to estradiol-treated cells as the positive control group (p<0.001) (Figure 3b). Taken together, these results revealed that the osteogenic activity of *F. foetida* was greater than *F. szowitsiana* and *F. flabelliloba*. 

***Runx-2*** **gene expression**

**Figure 4:**
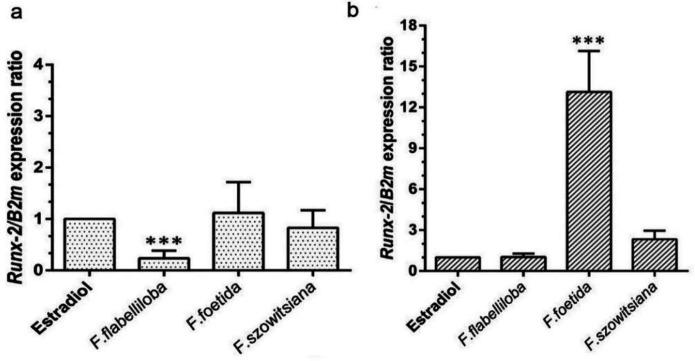
*Runx-2* gene expression. The expression of *Runx-2* gene was measured in MenSCs treated with 20 µg/ml of different plant extract including *F. flabelliloba*, *F. foetida*, and *F. szowitsiana*, and estradiol (10^-7^ M, positive control) (a) 14, and (b) 21 day after the treatment. The expression of *Runx-2* was normalized against the endogenous control (*B2m*).All data are expressed as mean±SD (***p<0.001)

Bone formation activity of the plant extracts was further assessed by *Runx*-*2* gene expression analysis 14 and 21 days after treatment using qRT-PCR method. The results highlighted that after 14 days of differentiation induction, *F. szowitsiana and F. foetida *had the same stimulatoryeffects on the *Runx-2* expression level as estradiol (Figure 4a). However, as shown in Figure 4b, after 21 days of treatment, *Runx-2* was significantly up-regulated in the cells treated with *F. foetida* in comparison with estradiol-, *F. flabelliloba*-, and *F. szowitsiana*-treated cells (p<0.001).

## Discussion

In this study, we evaluated the osteogenic activity of *F. Flabelliloba*, *F. foetida*, and *F. szowitsiana*methanolic-dichloromethaneextracts on well-characterized human menstrual blood-derived mesenchymal stem cells in an *in vitro* model. The most striking evidence from the data was that the extract of *F. szowitsiana* and *F. foetida* exerted positive effects on human MenScs osteogenesis and osteoblastic marker similar to 17β-Estradiol. 

*Ferula *spp*.* extract has been widely used in folklore medicine in Iran and is a rich source of phytochemicals that have beneficial effects on human health (Akaberi et al., 2015 ▶). The similar effects of phytoestrogen on bone mass compared to the estradiol (Palumbo et al., 2012 ▶; Zavatti et al., 2013 ▶) persuaded us to investigate the estrogen-like activity of different *Ferula *spp*.* extracts *in vitro*. 

Initially, the toxic concentration of the plant extracts was determined. The extract of *F. foetida* at high concentrations (50, 75 and 100 µg/ml) stimulated the proliferation of MenSCs and its cytotoxic effects emerged at low concentrations. Conversely, the proliferation of MenSCs exposed to high concentration of *F. flabelliloba* extract decreased. A similar effect has been reported for other phytoestrogens such as Fennel extract (Mahmoudi et al., 2013 ▶). Based on IC50 values, 20 µg/ml of each plant extract was selected to examine and compare their osteogenic activity. 

To further confirm the osteogenesis stimulatory effect of the plant extracts, we assessed the ALP activity and the expression of *Runx-2* as a specific osteoblastic marker of osteogenesis, on day 14 and 21 post-treatment. ALP activity, the well-recognized and early osteoblastic marker, plays a considerable role in the initiation of mineralization process during bone formation (Golub and Boesze-Battaglia, 2007 ▶). Increased ALP activity was observed in the MenSCs treated with *F. foetida*, and *F. szowitsiana* after 21 days in comparison with the vehicle control group (DMSO) while *F. Flabelliloba* extract could not induce ALP activity. Our experiments are in line with previous results (Mahmoudi et al., 2013 ▶), which examined the role of ethanolic extract of *Ferula gummosa* on MSCs osteogenic differentiation and demonstrated that these cells presented significant up-regulation of ALP activity. 

The level of *Runx-2 *mRNAwas determined after 14 and 21 days of treatment with different plant extracts. *Runx-2* transcription factor is a positive regulator of osteoblast differentiation by stimulating the expression of proteins such as osterix and osteocalcine which are necessary for bone formation and development (Ducy et al., 1997 ▶; Zainabadi et al., 2017 ▶). According to our data, *Runx-2* expression in the presence of *F. foetida* was similar to those treated with 17β-estradiol for 14 days treatment. The greater expression of this marker was observed after 21 days of exposure to *F. foetida* or *F. szowitsiana* compared to 17β-estradiol. Our findings are in the same direction as those reported by Geoffroy et al. and Liu et al. (Geoffroy et al., 2002 ▶; Liu et al., 2001 ▶). They demonstrated that *in vivo* overexpression of Runx-2 can enhance and be sufficient for osteoblast differentiation activation. Our results unexpectedly revealed that the ALP activity is not necessarily correlated with *Runx2* gene expression (Figures 3b and 4) even though this result differs from an earlier study (Zhao et al., 2005 ▶), it is consistent with the previous findings (Komori et al., 1997 ▶) reported in the literature with regard to stimulation of ALP activity in Runx-2-deficient cells (*Runx-2*^-/-^). The expression of osteoblast marker genes such as alkaline phosphatase is not directly stimulated by levels of Runx2 (Franceschi et al., 2003 ▶). There is a possibility that Dlx5 may stimulate ALP promoter activity in a Runx-2-independent manner (Kim et al., 2004 ▶). Further data would be needed to determine exactly how the expression of ALP and other osteoblast related genes are regulated by the plant extracts.

In conclusion, our study suggests that *F. foetida and F. szowitsiana *have stimulatory effects on osteogenesis. Thisfurther highlights the medicinal values of these plant extracts in bone repair or prevention of osteoporosis. However, more experiments in animal models and clinical studies are required to confirm these potential therapeutic benefits.
